# Fine-Mapping the Genetic Association of the Major Histocompatibility Complex in Multiple Sclerosis: HLA and Non-HLA Effects

**DOI:** 10.1371/journal.pgen.1003926

**Published:** 2013-11-21

**Authors:** Nikolaos A. Patsopoulos, Lisa F. Barcellos, Rogier Q. Hintzen, Catherine Schaefer, Cornelia M. van Duijn, Janelle A. Noble, Towfique Raj, IMSGC, ANZgene, Pierre-Antoine Gourraud, Barbara E. Stranger, Jorge Oksenberg, Tomas Olsson, Bruce V. Taylor, Stephen Sawcer, David A. Hafler, Mary Carrington, Philip L. De Jager, Paul I. W. de Bakker

**Affiliations:** 1 Program in Translational NeuroPsychiatric Genomics, Institute for the Neurosciences, Department of Neurology, Brigham & Women's Hospital, Boston, Massachusetts, United States of America; 2 Division of Genetics, Department of Medicine, Brigham & Women's Hospital, Harvard Medical School, Boston, Massachusetts, United States of America; 3 Harvard Medical School, Boston, Massachusetts, United States of America; 4 Broad Institute of Harvard and Massachusetts Institute of Technology, Cambridge, Massachusetts, United States of America; 5 Division of Epidemiology, Genetic Epidemiology and Genomics Laboratory, School of Public Health, University of California, Berkeley, Berkeley, California, United States of America; 6 Kaiser Permanente Division of Research, Oakland, California, United States of America; 7 Department of Neurology, MS Centre ErasMS, Erasmus MC, Rotterdam, The Netherlands; 8 Genetic Epidemiology Unit, Department of Epidemiology and Biostatistics and Clinical Genetics, Erasmus MC, Rotterdam, The Netherlands; 9 Children's Hospital Oakland Research Institute, Oakland, California, United States of America; 10 Department of Neurology, University, of California at San Francisco, San Francisco, California, United States of America; 11 Section of Genetic Medicine, Department of Medicine, University of Chicago, Chicago, Illinois, United States of America; 12 Institute for Genomics and Systems Biology, University of Chicago, Chicago, Illinois, United States of America; 13 Department of Clinical Neuroscience CMM, Karolinska Institutet, Stockholm, Sweden; 14 Menzies Research Institute Tasmania, University of Tasmania, Hobart, Australia; 15 University of Cambridge, Department of Clinical Neuroscience, Addenbrooke's Hospital, Cambridge, United Kingdom; 16 Department of Neurology and Department of Immunobiology, Yale University, School of Medicine, New Haven, Connecticut, United States of America; 17 Cancer and Inflammation Program, Laboratory of Experimental Immunology, SAIC Frederick, Frederick National Laboratory for Cancer Research, Frederick, Maryland, United States of America; 18 Ragon Institute of MGH, MIT, and Harvard, Charlestown, Massachusetts, United States of America; 19 Department of Medical Genetics, Division of Biomedical Genetics, University Medical Center, Utrecht, The Netherlands; 20 Julius Center for Health Sciences and Primary Care, University Medical Center, Utrecht, The Netherlands; Georgia Institute of Technology, United States of America

## Abstract

The major histocompatibility complex (MHC) region is strongly associated with multiple sclerosis (MS) susceptibility. *HLA*-DRB1*15:01 has the strongest effect, and several other alleles have been reported at different levels of validation. Using SNP data from genome-wide studies, we imputed and tested classical alleles and amino acid polymorphisms in 8 classical human leukocyte antigen (HLA) genes in 5,091 cases and 9,595 controls. We identified 11 statistically independent effects overall: 6 *HLA-DRB1* and one *DPB1* alleles in class II, one *HLA-A* and two *B* alleles in class I, and one signal in a region spanning from *MICB* to *LST1*. This genomic segment does not contain any HLA class I or II genes and provides robust evidence for the involvement of a non-HLA risk allele within the MHC. Interestingly, this region contains the *TNF* gene, the cognate ligand of the well-validated *TNFRSF1A* MS susceptibility gene. The classical HLA effects can be explained to some extent by polymorphic amino acid positions in the peptide-binding grooves. This study dissects the independent effects in the MHC, a critical region for MS susceptibility that harbors multiple risk alleles.

## Author Summary

Multiple sclerosis (MS) is an inflammatory and neurodegenerative disease with a heritable component. Although it has been known for a long time that the strongest MS risk factor maps to the major histocompatibility complex (MHC) on chromosome 6, there are still many unresolved questions as to the identity and the nature of the risk variants within the MHC. Because the MHC has a complex structure, systematic investigation across this region has been challenging. In this study, we used state-of-the-art imputation methods coupled to statistical regression to query variants in the human leukocyte antigen (HLA) class I and II genes for a role in MS risk. Starting from available SNP genotype data, we replicated the strongest risk factor, the HLA-DRB1*15:01 allele, and were able to identify 11 independent effects in total. Functional studies are now needed to understand their mechanism in MS etiology.

## Introduction

Across the entire human genome, the major histocompatibility complex (MHC) on chromosome 6 makes the single largest contribution to multiple sclerosis (MS) susceptibility. The classical HLA-DRB1*15:01 allele has been documented as the strongest association to MS risk, and its role has been studied and replicated extensively [1]. Numerous other HLA alleles have been suggested to be associated with MS susceptibility, but the complex structure of the MHC has made it challenging to unequivocally pinpoint variants that play a causal role in MS [1], [2]. For example, it has been suggested that DQB1*06:02, an MHC class II allele in strong linkage disequilibrium (LD) with DRB1*15:01, either has no independent effect [3] or acts in an extended haplotype with DRB1*15:01, the DRB1*15:01—DQB1*06:02 haplotype, or the DRB1*15:01—DQA1*0102—DQB1*06:02 haplotype [4], [5]. The ambiguity and the lack of replication for many of the MHC associations can be attributed to the extended LD structure of the MHC [6], the limited number of HLA loci analyzed, and the relatively small sample size of previous studies.

Thanks to a large sample size and a novel procedure to impute classical HLA alleles from SNP data, a recent study described independent MHC effects for DRB1*15:01, *03:01 and *13:03 as well as *HLA*-A*02:01 and rs9277535 [7]. In the present study we sought to test not only the role of classical HLA alleles but also of potentially functional variation within the HLA genes. To this end, we imputed classical alleles as well as their corresponding amino acid sequences in 8 HLA genes in a large population of 5,091 MS cases and 9,595 healthy controls, with genome-wide data (GWAS), following a recently described imputation protocol [8]. Both the samples and the imputation method used were independent of recent efforts exploring MHC associations to MS susceptibility [7].

## Results

We have successfully imputed 3,613 SNPs, 202 amino acid positions, 78 classical HLA alleles at two-digit resolution, and 99 classical HLA alleles at four-digit resolution (Figure S1). Given the number of hypotheses that are tested in this analysis, we set, *a priori*, p<1×10^−5^ as the threshold for statistical significance. This threshold accounts for 5,000 independent tests, assuming a study-wide type 1 error rate (α) of 5%. Overall, we analyzed 5,091 MS cases and 9,595 healthy controls from eight different GWAS data sets (Table 1).

**Table 1:** Descriptive characteristic of analyzed data sets.

GWAS	Cases	Controls	%males	Genomic inflation factor
GeneMSA DU	219	225	39%	1.020
GeneMSA US	437	402	32%	1.021
GeneMSA SW	239	190	30%	1.018
IMSGC	790	1677	43%	1.030
BWH	821	2705	51%	1.058
ANZgene	1582	1949	33%	1.054
Rotterdam	459	1938	41%	1.030
Kaiser Permanente	544	513	19%	1.003

IMSGC: International Multiple Sclerosis Genetics Consortium; BWH: Brigham & Women's Hospital; ANZ: Australia and New Zealand genetic study; DU: Netherlands; US: United States; SW: Switzerland.

### Multi-allelic nature of association at HLA-DRB1

**Figure 1:**
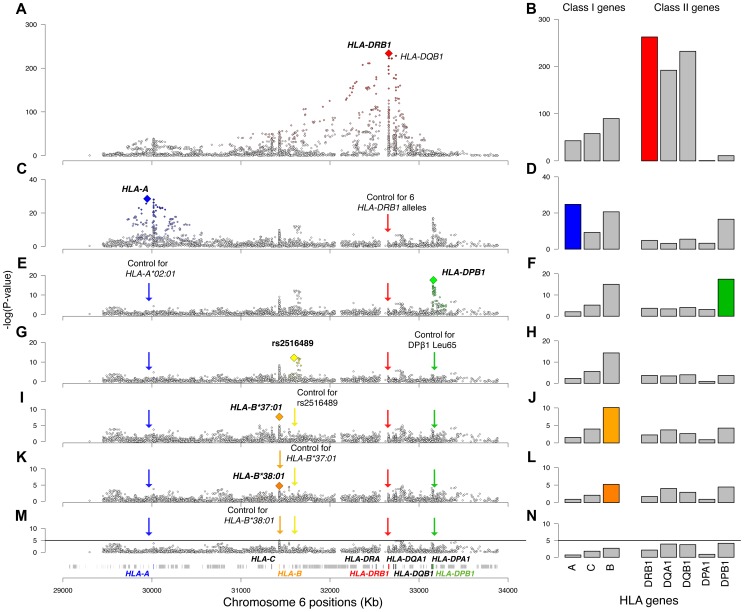
Association plots for the analyzed variants and HLA genes. Panels on the left are regional association plots for all variants in the MHC region; each diamond is a polymorphism evaluated in the analysis (SNP, HLA type, amino acid). The index variant that best captures the effect of each locus is highlighted with a larger diamond. Variants that are in LD with the index variant are colored, with the intensity of the color being proportional to the extent of LD. The panels on the right plot the −log_10_(p-value) of the eight analyzed HLA genes. The order of the genes is based on their position on chromosome 6. The rows represent univariate analysis (A, B), conditioning on six *HLA-DRB1* alleles (*15:01, *03:01, *13:03, *04:04, *04:01, and *14:01) (C, D), conditioning on the above and HLA-A*02:01 (E, F), conditioning on the above and the *DPB1* effect (G, H), conditioning on the above and rs2516489 (I, J), conditioning on the above and B*37:01 (K, L), conditioning on the above and B*38:01 (M, N). In panels M and N the solid black line marks the threshold of statistical significance in the study.

The most statistically significant variant in the univariate analysis (see Material and Methods for details) was HLA-DRB1*15:01 (odds ratio [OR] = 2.92, p = 1.4×10^−234^, Figure 1A). Looking at each category of variants (SNPs, two-digit HLA alleles, four-digit HLA alleles and amino acid positions), the amino acid position with the smallest p-value was position −5 in the leader peptide of DQβ1 (p = 7.6×10^−231^), and the most statistically significant SNP was at position 32,742,280 (OR for the A allele = 2.96, p = 5.1×10^−229^). An equivalent effect was observed for HLA-DQB1*06:02 (OR = 2.96, p = 5.4×10^−229^). We first tested whether the DRB1*15:01 effect could be explained by *DQB1*. Adjusting for *DQB1* variants, we observed that DRB1*15:01 always had a residual effect (p<10^−6^). Conversely, adjusting for DRB1*15:01, the effect of *DQB1* variants were accounted for (p>0.8), suggesting that DRB1*15:01 had a non-equivalent and more statistically significant effect than the *DQB1* variants. Furthermore, the extended DRB1*15:01—DQB1*06:02 haplotype (p = 7.5×10^−231^) did not improve upon the association of DRB1*15:01 alone. Similarly, the classical DQA1*01:02 allele—that was suggested to contribute to the effect of the haplotype—was strongly associated (p = 4.8×10^−178^), but its effect could be entirely explained by DRB1*15:01. These observations strengthen the hypothesis that the primary MHC effect in MS is mediated by DRB1*15:01 and not by variants in the *DQB1* or *DQA1* loci.

The *DRB1* locus (all four-digit alleles in one model) had a p-value of 4.0×10^−263^ in the initial analysis (Figure 1B). After adjusting for DRB1*15:01, the residual *DRB1* locus effect (due to all remaining *DRB1* four-digit alleles) was still statistically significant (p = 3.1×10^−37^), indicating the presence of multiple independent *DRB1* effects. Applying a forward stepwise strategy (see Materials and Methods for details), we established statistical independence for 5 additional *DRB1* alleles: *03:01, *13:03, *04:04, *04:01, and *14:01 (Table S1). After controlling for the effects of all 6 significant *DRB1* alleles (including *15:01), there was no evidence for a residual signal (p = 1.5×10^−05^). We also applied several other variant selection approaches to test the robustness of these findings; all approaches identified the same six alleles (Table S1).

### HLA-A*02:01 has an independent protective effect

**Figure 2:**
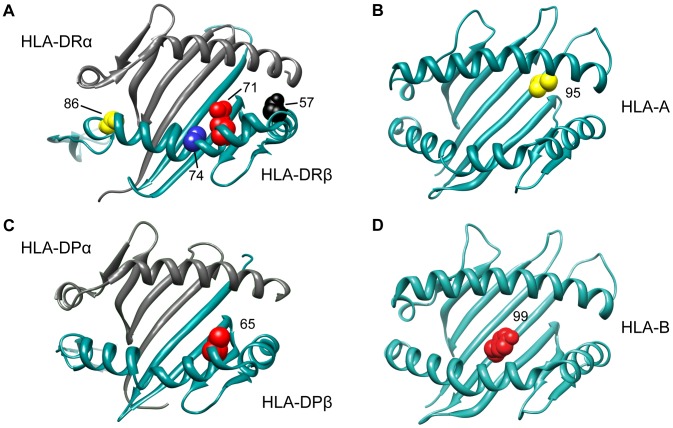
3D ribbon models for HLA DR, HLA A, HLA DP and HLA B. All structures are positioned to accommodate the view of the peptide-binding groove and the associated amino acid residues. The Protein Data Bank entries 3pdo, 1a1m, 3lqz, and 2bvp were used to produce the 3D structures, respectively, using UCSF Chimera [49].

Having analyzed the effects at *HLA-DRB1*, we tested all other variation across the MHC while correcting for the six statistically independent *DRB1* alleles, namely DRB1*15:01, DRB1*03:01, DRB1*13:03, DRB1*04:04, DRB1*04:01, and DRB1*14:01. The most statistically significant variant was SNP rs2844821 near *HLA-A* (OR for G allele = 0.70, p = 3.2×10^−29^, Figure 1C). Due to LD, this SNP effect is statistically equivalent to the effect of HLA-A*02:01 (OR = 0.70, p = 7.4×10^−29^) and amino acid Val at position 95 in the peptide-binding groove of the HLA-A protein (OR = 0.70, p = 9.6×10^−29^, Figure 2). Controlling for this effect, there were no other *HLA-A* associations.

### The DPB1 association with MS susceptibility

Controlling for the 6 *DRB1* alleles and the *HLA-A* effect, the next most statistically significant variant was rs9277489 (OR for C = 1.31, p = 2.6×10^−18^). This SNP is in the intronic region of *HLA-DPB1* gene and in perfect LD (r^2^ = 1, based on HapMap Phase II) with rs9277535 that was previously associated with MS susceptibility [7], [9]. The most statistically significant HLA allele was DPB1*03:01 (p = 3.6×10^−15^), but the effect of rs9277489 cannot be explained by DPB1*03:01 alone (p = 1.7×10^−06^ for rs9277489 in the presence of DPB1*03:01). The most statistically significant amino acid mapped to position 65 of HLA-DPβ1 (OR for Leu vs. Ile = 1.37, p = 3.7×10^−18^), which explained the effect of rs9277489 (p = 0.003 for rs9277489 in the presence of Leu65 in HLA-DPβ1). This amino acid is also located in the peptide-binding groove of HLA-DPβ1 (Figure 2). After controlling for rs9277489, there was no residual effect at the *DPB1* locus (p>1.0×10^−5^).

### A non-classical MHC association in MICB-LST1

**Figure 3:**
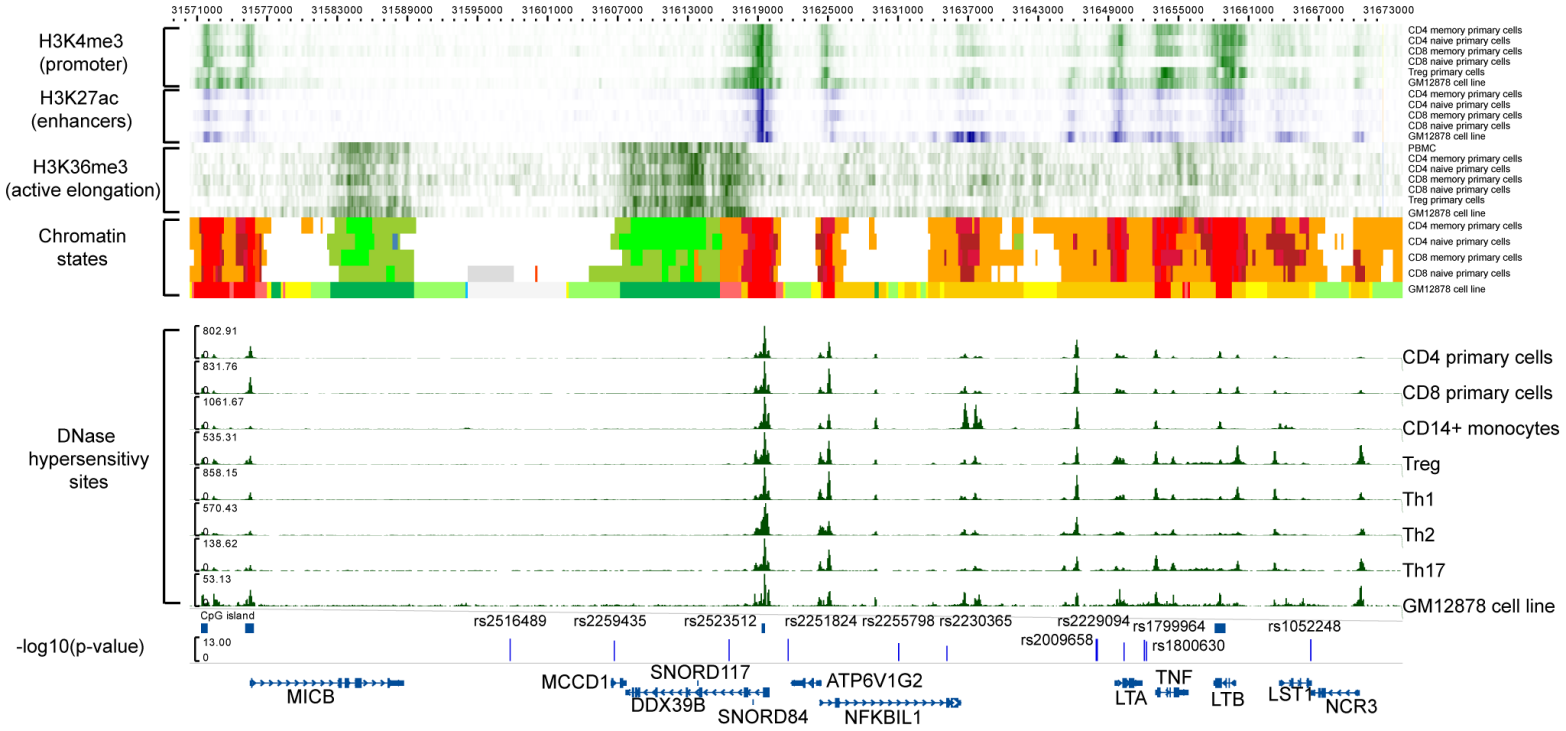
Functional annotation of the *MICB-LST1*. The first 6 rows are H3K4me3 (green) data for CD4 memory primary cells, CD4 naïve primary cells, CD8 memory primary cells, CD8 naïve primary cells, Treg primary cells, and GM12878 cell line (B-lymphocyte, lymphoblastoid, International HapMap Project - CEPH/Utah - European Caucasion, Epstein-Barr Virus). The next 5 rows display H3K27ac (blue) data for CD4 memory primary cells, CD4 naïve primary cells, CD8 memory primary cells, CD8 naïve primary cells, and GM12878 cell line, respectively. Then there are the H3K36me3 (green) data for CD4 memory primary cells, CD4 naïve primary cells, CD8 memory primary cells, CD8 naïve primary cells, Treg primary cells, and GM12878 cell line. The chomatin states displayed are for CD4 memory primary cells, CD4 naïve primary cells, CD8 memory primary cells, CD8 naïve primary cells, and GM12878 cell line. The DNase hypersensitivity sites are for CD4 primary cells, CD8 primary cells, CD14+ monocytes, Treg, Th1, Th2, Th17 and GM12878 cell line, respectively. The detailed colorscheme of the chromatin states is listed in the Supplementary material. Briefly, red corresponds to transcription start sites (TSSs) and/or active promoters, orange/yellow to enhancers, green to transcription, and white/grey to heterochromain. All data are publicly available data from ENCODE and NIH Roadmap. The last row displays the −log10(p) of the SNPs in the LD block after adjustment with the HLA-DRB1*15:01 effect.

Adjusting also for the *DPB1* effect, we identified rs2516489 as the next most statistically significant variant (OR for T = 1.31, p = 6.7×10^−13^, Figure 1G, Figure S2B). This SNP tags a region of extended LD containing several non-classical MHC class I, class III and cytokine genes, i.e. *MICB*, *DDX39B* (*BAT1*), *NFKBIL1*, *TNF*, *LTA*, *LTB*, and *LST1* (Figure 3). We note that this region had no substantial effect in the univariate analysis (Figure 1A, Figure S2A), but it became genome-wide significant once the DRB1*15:01 effect was accounted for (Table S2). There was no evidence of interaction either with DRB1*1501 (Table S2) or any other of the identified effects. To explore this phenomenon further, we stratified the samples according to the presence of DRB1*15:01 into carriers (heterozygous and homozygous) and non-carriers. Univariate analysis in these two strata revealed a consistent but modest effect (OR ∼1.2) for the associated SNP in both DRB1*15:01 carriers and non-carriers (Table S2, Figure S3). This phenomenon can likely be explained by Simpson's paradox, where two subgroups share the same association but the overall population shows no association (or even a reversed one) [10]. This analysis therefore returns, for the first time, robust evidence supporting the role of non-HLA genes within the MHC.

To explore any functional consequences of the SNPs in the *MICB-LST1* region we tested these SNPs for *cis*-eQTL (expression quantitative trait loci) effects in peripheral blood mononuclear cells (PBMCs) of 213 MS subjects [11] as well as CD4+ T cells and CD14+ monocytes of 211 healthy controls (Table S3). None of the associated SNPs had a strong *cis*-eQTL effect (p>1×10^−5^): the strongest effect in this region is the relation of rs2516489 to *LST1* expression (p = 1.91×10^−5^) in the CD4+ T cells of healthy individuals. The next strongest effect also involved rs2516489 but was seen in relation to *HCG18* (p = 3.19×10^−5^) in the PBMCs of MS subjects. None of the SNPs had a statistically significant *cis*-eQTL effect on any of the class I or II classical HLA genes (Table S3). Leveraging the publicly available Encyclopedia of DNA Elements (ENCODE) [12] and NIH Epigenomics Roadmap [13] for immune cells and cell lines it is evident that the region has an abundance of functional elements (Figure 3). Of specific interest is the non-coding naturally occurring read-through transcription between the neighboring *ATP6V1G2* (ATPase, H+ transporting, lysosomal 13 kDa, V1 subunit G2) and *DDX39B* (DEAD box polypeptide 39B) genes. Two SNPs, rs2523512 and rs2251824, tag this element that has a strong signal in the DNase hypersensitivity assay in all immune cell types, suggesting that it is an active *cis*-regulatory region. The histone markers for promoters, enhancers and active elongation also support these data, while this region is identified as an active transcription start site using chromatin states [14]. Other candidates are the *TNF* and *LTB* genes. Rs2516489, the SNP with the best (but not statistically significant) *cis*-eQTL effects, lies within a region of heterochromatin, with no indication of regulatory potential in the available data.

### Independent HLA-B effects

Adjusting for 6 classical *DRB1* alleles, HLA-A*02:01, rs9277489 (*HLA-DPB1* effect) and rs2516489, we observed another novel signal emerging from the *HLA-B* locus (p = 7.9×10^−11^). The most statistically significant variants were HLA-B*37, HLA-B*37:01, amino acid Ser at position 99 in HLA-B (Figure 2) and a SNP in position 31,431,006 (hg18) (Figure 1I, J). All of these variants had statistically equivalent effects (OR = 1.75, p = 2.2×10^−08^). Accounting for the effect of HLA-B*37:01, no other variant in *HLA-B* exceeded our *a priori* defined threshold, although the residual effect at the *HLA-B* locus due to all remaining classical *HLA-B* alleles was still statistically significant in our analysis (p = 6.5×10^−06^, Figure 1L). This residual association could be accounted for by HLA-B*38:01 (OR = 0.55; p = 4.1×10^−05^). After adding HLA-B*38:01 to the model, there was no longer evidence for a residual effect of classical *HLA-B* alleles (p>0.002) or elsewhere across the MHC. No amino acid position in HLA-B could explain the HLA-B*38:01 effect.

### Amino acid residues in DRβ1

Next, we set out to assess whether a specific set of amino acids within the HLA-DR molecule could explain the collective effect of the six classical *DRB1* alleles identified above. To this end, we tested each polymorphic amino acid position using an omnibus test (a regression model with all but one amino acids carried by a given position), adding all amino acids (but one) of the most statistically significant position to the model in a forward stepwise fashion. The most significant amino acid position in DRβ1 mapped to position 71 (p = 1.2×10^−227^, Figure S4A), which carries 4 possible alleles: Ala, Arg, Glu, and Lys. Controlling for the alleles at position 71 (df = 3), there was still a strong residual signal for DRB1*15:01 (p = 5.8×10^−13^), indicating that amino acid position 71 alone does not explain the DRB1*15:01 effect. Adjusting for the alleles at position 71, position 74 was the next most statistically significant (p = 1.2×10^−16^, Figure S4B). This position harbors five possible alleles: Arg, Leu, Glu, Ala and Gln. Controlling for positions 71 and 74, position 57 (with four alleles: Asp, Ser, Val or Ala) was the next most statistically significant association (p = 4.9×10^−11^, Figure S4C). Controlling for positions 71, 74 and 57, we found position 86 as the most statistically significant association (OR = 1.35 for Val vs. Gly, p = 1.0×10^−06^, Figure S4D). After controlling for these four positions, no other amino acid position exceeded our significance threshold (Figure S4E), although HLA-DRB1*15:01 still showed a residual association signal (p = 10^−05^). The model with the four DRβ1 amino acid positions could explain the data better than a model with only DRB1*15:01 (p = 2.6×10^−26^ in favor of the DRβ1 amino acid positions), but it was slightly worse than the model with the six *DRB1* alleles (p = 0.001 in favor of the 6 *DRB1* alleles). All four amino acid positions reside in the peptide-binding groove of the HLA-DR molecule (Figure 2; Table S4 lists the correspondence between the amino acids at these positions and the six associated classical *DRB1* alleles).

### Variance explained

Integrating all of the results, HLA-DRB1*15:01 accounted for 10% of the phenotypic variance in the data, whereas all 6 independent *HLA-DRB1* alleles explained 11.6%. A model with all identified statistically independent effects (HLA-DRB1*15:01, HLA-DRB1*03:01, HLA-DRB1*13:03, HLA-DRB1*04:04, HLA-DRB1*04:01, HLA-DRB1*14:01, HLA-A*02:01, rs9277489/Leu65 in HLA-DPβ1, rs2516489, HLA-B*37:01, and HLA-B*38:01) accounted for 14.2% of the total variance in MS susceptibility.

## Discussion

We have imputed classical alleles of HLA genes, their corresponding amino acids and SNPs across the MHC, and tested all variants for association in a large case-control collection. Our analysis corroborates the effects of *DRB1* alleles other than the well-known DRB1*15:01 association. Classical alleles DRB1*03:01, *13:03, *04:04, *04:01, and *14:01 display robust, independent associations in our data. The *DQB1* and *DQA1* genes have been suggested to form extended haplotypes with *DRB1* alleles, mostly *15:01 [4]. In our hands, the effect of DQB1*06:02 does not explain the effect of DRB1*15:01. Furthermore, the DRB1*15:01—DQB1*06:02 haplotype does not appear to explain the data as well as the effect of DRB1*15:01 alone. Based on these results, DRB1*15:01 and the remaining *DRB1* alleles are better candidates than *DQB1* variants for a causal role in MS susceptibility, a hypothesis that agrees with the MHC analysis of MS subjects with African origin [3]. We note that this interpretation counters evidence in favor of *DQB1* from certain murine models that capture elements of human inflammatory demyelination by triggering experimental autoimmune encephalomyelitis induced with myelin-associated oligodendrocytic basic protein [15] or proteolipid protein [16].

A number of studies have highlighted the importance of class I HLA alleles in MS susceptibility, with HLA-A*02:01 being the most prominent allele [17]–[20]. Here, we replicated the HLA-A*02:01 association and attributed it to an amino acid polymorphism at position 95 in the peptide-binding groove of the HLA-A molecule. We also replicated the recently proposed DPB1*03:01 association, and identified a more statistically significant effect at amino acid position 65 in the peptide binding groove of HLA-DPβ1 [7], [9]. Although our study has overlapping samples with the first study to identify an independent *HLA-DPB1* effect [9], these account for only 24% of the present sample set. The evidence of an *HLA-DPB1* effect is strengthened by the fact that the second study reporting such a signal [7] has no overlapping samples with our study. Furthermore, we confirmed the presence of statistically independent *HLA-B* effects [21], [22]. Our analysis fine-mapped these to B*37:01 and B*38:01. Of these, B*37:01 can be explained by amino acid Ser99 of the HLA-B protein, which is also in this molecule's peptide-binding groove. The *HLA-C* locus demonstrated no convincing evidence for a statistically independent effect, suggesting that previous results may have tagged untested *HLA-A* or *HLA-B* effects across the class I region [23]. Although some of the above associations could be explained by specific amino acid polymorphisms in the corresponding HLA proteins, the picture at *HLA-DRB1* however appears to be more complex as there was no single model based on amino acids that could explain the entire locus effect (including the specific effect due to DRB1*15:01). At this stage, our conservative interpretation of these results is that the implicated amino acids allow new hypotheses to be formulated for future functional studies.

An interesting finding in our analysis was the association of the region spanning from *MICB* to *LST1*, which contains several important class I, class III and cytokine-related genes. Although the identified SNPs were not significant in the initial (univariate) analysis, we established that these reached significance after adjusting for the strong DRB1*15:01 effect. One small study previously examined *MICB* along with DRB1*15 and had found evidence for an independent association [24]. Another study reported that variation in *TNF* can modify the effect of DRB1*15:01 [25]. We did not obtain evidence for statistical interaction between this locus and the other MHC variants, indicating that the MHC susceptibility variants we have catalogued likely act independently and additively in terms of MS susceptibility. Overall, we offer robust evidence for the role of a specific MS susceptibility haplotype in this region of the MHC. This region harbors evidence for association with several other diseases, e.g. Crohn's disease and ulcerative colitis [26], rheumatoid arthritis [27], Sjogren's syndrome [28], and hepatitis C virus-associated dilated cardiomyopathy [29]. However, the identity of the causal gene(s) within this associated region remains unclear at this time, but it is intriguing that three of the genes (*TNF*, *LTA* and *LTB*) are ligands for one of the validated MS susceptibility genes, *TNFRSF1A* [30]. We did not observe any evidence of statistical interaction (p>0.5) with this non-MHC locus in our data. Our preliminary analysis using *cis*-eQTL data in healthy individuals and MS subjects as well as the publicly available genomic data from the ENCODE and NIH Epigenomics Roadmap did not identify a single variant/gene as the likely causal one. From this information it seems that several genes have functional potential, but more detailed functional studies will be needed to unravel the causal variants and genes.

Leveraging genome-wide genotype data, the collection of analyses presented here provides a well-powered investigation of thousands of genotyped and imputed SNPs, classical alleles of 8 class I and II HLA genes and amino acid sequence variation of these HLA proteins. The combination of the large sample size with additional variation types allowed us to present an enhanced dissection of the critical role of the MHC in MS susceptibility. Our results highlight a possible role for certain residues in the peptide-binding groove of HLA molecules associated with peptide antigen recognition. In HLA-DRβ1 we identified a set of four amino acids in positions 71, 74, 57 and 86 that capture most (but not all) of the *DRB1* association. Of these, Val86 has been associated previously with MS [31]–[33], and this residue appears to be important for the presentation of peptides from a putative target antigen in MS, myelin basic protein [34], and for the stability of the DRαβ dimer [35]. Another study suggested an association at position 60 [36] and another one at position 13 [37], although these were not replicated in the present study. Interestingly, the HLA-DRβ1 amino acids in positions 71 and 74 were recently also associated with susceptibility to rheumatoid arthritis [38]. Overall, consistent with the known biology of MS, it appears that disease-associated variants in *HLA-DRB1* primarily influence the structural characteristics of the peptide-binding groove and presumably lead to alterations of the T cell repertoire that enhance the likelihood of an inflammatory demyelinating process. However, the MHC also harbors at least one other risk allele that does not directly affect an antigen-presenting molecule: the robust evidence supporting a risk haplotype in the vicinity of *MICB* will have a different mechanism, one that is likely to affect the function of one or perhaps several cytokines.

This study displays an effective strategy for in-depth characterization of this complex region of the human genome. Increasing study sample sizes and more complete reference panels are likely to continue to provide a more detailed perspective on the architecture of genetic susceptibility in this region. The identified amino acid residues may help prioritize the identification of binding peptides and investigations of other potential roles that these susceptibility alleles might have in the biology of MS susceptibility aside from antigen presentation.

## Materials and Methods

### Samples

We used data from 8 genome-wide association studies (GWAS) of European ancestry (Table 1): (a) three GWAS of the GeneMSA [30], [39] with samples from the Netherlands (GeneMSA DU), Switzerland (GeneMSA SW), and the United States (GeneMSA US); (b) an early GWAS from the IMSGC [30], [39], [40] with samples from the United States (ISMGC US) and the United Kingdom (IMSGC UK), that was collapsed in one stratum removing the UK cases; (c) a GWAS with cases from the Brigham and Women's Hospital and controls from the MIGEN study (BWH) [30], [39]; (d) the Australia and New Zealand Multiple Sclerosis Genetics Consortium (ANZgene) [41]; (e) an unpublished GWAS set from Erasmus Medical Center in Rotterdam, the Netherlands; and (f) an unpublished GWAS collection from the Kaiser Permanente MS Research Program (Kaiser Permanente). All the above GWAS data sets were filtered with the same quality control criteria as part of an ongoing meta-analysis of Multiple Sclerosis GWAS. In each of these data sets we performed principal components analysis (PCA) to identify population outliers and to calculate covariates to control for population stratification between cases and controls.

### Imputation of classical HLA type I and II alleles and respective amino acid sequences

From each GWAS we extracted SNPs within the extended MHC region (chr6:29,299,390 to 33,883,424; hg18) to impute classical alleles for class I HLA genes (*HLA-A*, *HLA-B*, and *HLA-C*) and class II HLA genes (*HLA-DPA1*, *HLA-DPB1*, *HLA-DQA1*, *HLA-DQB1*, and *HLA-DRB1*), their corresponding amino acid sequences and SNPs not captured in the genotypic platforms used. The imputation was performed with the software BEAGLE [42] using a collection of 2,767 individuals of the Type 1 Diabetes Genetics Consortium (T1DGC) with 4-digit classical allele genotyping for the above HLA genes as the reference panel. This method and reference panel have been used for fine-mapping MHC associations in HIV control [8] and seropositive rheumatoid arthritis [38]. Cases and controls from each GWAS dataset were imputed together. All variants in the reference panel were coded as biallelic markers (presence vs. absence), allowing us to use BEAGLE for the imputation. Post-imputation we excluded variants with minor allele frequency less than 1% from the analysis. Table S5 lists the imputation quality for the identified variants.

### Statistical analysis

We analyzed each variant using a logistic regression model, assuming alleles have an additive effect on the log-odds scale. We also assumed the genetic effects were fixed across all eight GWAS. In each model we included the top 5 principal components to control for within-GWAS population stratification and 7 dummy variables to account for between-GWAS specific effects. Throughout the text we refer to such a model as *univariate* (*M_u_*), even if several covariates were included in the model, reflecting the fact that only one MHC-specific variant is included in the model. This is the representation of the univariate model:
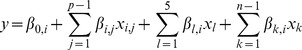
(1)
*M_u_*, Univariate logistic regression model

where *y* is the log(odds) for the case-control status, *β_0_* is the logistic regression intercept and *β_i,j_* is the log-additive effect for the allele *j* of the variant *i* with *p* alleles. In this paper, the term variant is used for any type of SNP (biallelic, triallelic, etc), two-digit HLA allele, four-digit HLA allele and amino acid position. In any case we included *p-1* alleles, with the one excluded being the reference allele. Where possible we tried to select the most frequent variant in the controls as the reference allele. The five included principal components are represented in the model as *l* and the last block in the model represents the dummy variables included for the *n* studies (*n-1* parameters added in the model).

To calculate an omnibus p-value for the variant, regardless of the number of alleles included in the univariate model, we used using a log-likelihood ratio test (2) comparing the likelihood *L_0_* of the null model (3) against the likelihood *L_1_* of the fitted model:
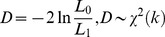
(2)Log-likelihood ratio test

where *D* is the log-likelihood test value, also known as deviance. *D* follows an approximate chi-square distribution with *k* degrees of freedom, where *k* is the difference of the regressed parameters between the two models. Representation of the null model:
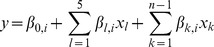
(3)
*M_0_*, Null logistic regression model

Besides testing variants for association, i.e. SNPs, HLA alleles and amino acids, we also fitted models that estimated the overall effect of the each of the eight HLA genes. We did so, by fitting all respective four-digit alleles of a given HLA gene in the same model. The respective p-values reflect the overall significance of the gene.

### Framework for identification of statistically independent effects

In order to identify the statistically independent effects, we first tested all variants under a univariate logistic regression model and ranked them based on the p-value of the log-likelihood test. Next, in a forward stepwise fashion, we included in the logistic regression model the most statistically significant variant as a covariate, analyzed all remaining variants and ranked them based on the new p-value of the respective log-likelihood test. The models that included at least one variant as covariate are referred to as *conditional* throughout the text. In each iteration the null model used in the log-likelihood test was the original null model (3) with the variants that were used as covariates. We repeated the same steps until no variant or no HLA gene reached the level of statistical significance, which we *a priori* set to be 10^−5^. This statistical significance threshold accounts of 5,000 independent tests using Bonferroni correction. Although most of the variants analyzed are correlated, we chose this threshold to account also for the multiple stepwise fitted models. If no variant reached the level of significance but an HLA gene did, we kept adding variants in the overall model until the HLA gene p-value was larger than 10^−5^.

To compare the effects of two (or more variants), e.g. A and B, we fitted the following models: *M_A_* model with variant A, *M_B_* model with variant B, and *M_AB_* model with both variants A and B. All three model included the same other covariates. Then we used the log-likelihood test to compare *M_AB_* vs. *M_B_* and *M_AB_* vs. *M_A_*. These two comparisons represent the effects of variants A and B, respectively, in the presence of the other variant, i.e. B and A. For these comparisons we used the nominal (α = 0.05) level of statistical significance.

### Statistically independent effects in the DRB1 locus

After adjusting for the most statistically significant variant, DRB1*15:01, the residual effect of the DRB1 locus, i.e. the effect of all alleles besides *15:01, was still the most statistically significant of any of the remaining variants. This led us to the hypothesis that several other DRB1 alleles could explain the overall DRB1 locus effect, already conditioning on DRB1*15:01. To identify such effects inside the DRB1 locus, we applied the above forward stepwise logistic regression approach to the four-digit DRB1 alleles. , To test the robustness of the results from the forward stepwise regression, we also applied four other statistical methods for variant selection: i) lasso, [43] ii) elastic net, [44] iii) least angle regression, [45] and iv) forward Stagewise regression. [46] For the lasso and elastic net we selected the largest value of lambda (*l_1_*) after 10-fold cross-validation, such that error was within 1 standard error of the minimum mean cross-validated error. In the respective results section, we illustrate that all methods reached the same conclusion independently.

### DRB1*15:01:DQB1*06:02 extended haplotypes (diplotypes)

It has been proposed that extended DRB1*15:01–DQB1*06:02 haplotypes confer the risk for MS rather than individual HLA alleles. To test this hypothesis, we used the post-imputation phased data to estimate the DRB1*15:01–DQB1*06:02 diplotypes. Then we fitted a logistic regression that estimated the effect of the diplotype under a per-allelic model. Since this approach used phased data, rather than post-imputation probabilities, the imputation uncertainty is not properly accounted for. Thus, we expect the respective p-values to be slightly inflated.

### Functional analysis of the MICB-LST1 region

To investigate the functional potential of the *MICB-LST1* region we queried:

- in-house cis-eQTL (expression quantitative trait loci) in PBMCs of 213 MS subjects [11] and CD4+ T cells and CD14+ monocytes of 211 healthy individuals. The PBMCs gene expression levels were quantified with mRNA derived from of 213 subjects of European ancestry with relapsing remitting (RR) multiple sclerosis (MS) via an Affymetrix Human Genome U133 Plus 2.0 Array. The expression levels were adjusted for confounding factors, such as subject's use of immunomodulatory drugs, age, gender, and batch effects via principle components analysis. Associations between SNP genotypes and adjusted expression residual traits were conducted by Spearman rank correlation (SRC). For the *cis* analysis, we considered only SNPs within a 2 Mbps window from the transcript start site (TSS) of genes. Furthermore, we also explored any possible effect to the expression of class I and II HLA classical genes, regardless of their physical distance. Significance of the nominal p-values was determined by comparing the distribution of the most significant p values generated by permuting expression phenotypes 10,000 times independently for each gene. We call a *cis*-eQTL significant if the nominal association p-value is greater than the 0.05 tail of the minimal p-value distribution resulting from the permuted associations, which corresponds to a p-value of 1×10^−05^. Similar methods were used to evaluate the *cis*-regulatory effects in CD4+ T lymphocytes and CD14+ monocytes data sets consisting of 211 healthy individuals of European ancestry. These analyses were conducted under the auspices of a protocol approved by the institutional review board of Partners Healthcare.
- publicly available data from the ENOCDE [12] and NIH Epigenomics Roadmap [13]. Specifically we perused data for functional potential from CD4 memory primary cells (H3K4me3, H3K27ac, H3K36me3, chromatin states), CD4 naïve primary cells (H3K4me3, H3K27ac, H3K36me3, chromatin states), CD8 memory primary cells (H3K4me3, H3K27ac, H3K36me3, chromatin states), CD8 naïve primary cells (H3K4me3, H3K27ac, H3K36me3, chromatin states), CD4 primary cells (DNase hypersensitivity sites), CD8 primary cells (DNase hypersensitivity sites), Treg primary cells (H3K4me3, H3K36me3, DNase hypersensitivity sites), Th1 (DNase hypersensitivity sites), Th2 (DNase hypersensitivity sites), Th17 (DNase hypersensitivity sites) and GM12878 cell line (B-lymphocyte, lymphoblastoid, International HapMap Project - CEPH/Utah - European Caucasion, Epstein-Barr Virus; H3K4me3, H3K27ac, H3K36me3, chromatin states, DNase hypersensitivity sites).

### Variance explained

We used Nagelkerke's pseudo-R [47] to estimate the variance explained
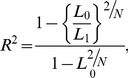
(4)Nagelkerke's pseudo-R^2^

where *L_0_* and *L_1_* are the likelihoods of the null model and fitted model respectively, and *N* is the number of individuals.

### Software

We used PLINK for the initial analysis of the data and to estimate minor allele frequencies and imputation quality metrics, i.e. INFO score. [48] We fitted all models in R using the glm function and package lars and glmnet.

### Ethics statement

This investigation has been approved by the Institutional Review Board of Partners Healthcare; the reference number is 2002p000434.

## Supporting Information

Figure S1 Scatter plots of minor allele frequency (MAF) and INFO score for the imputed variants. INFO score is an imputation quality metric and is defined as the ratio of the variance observed over the variance expected. On average genotyped SNPs have a value ∼1.
(PDF)

Figure S2 Regional associational plots for the non-classical HLA region spanning MICB-LST1. The figure displays the minus logarithmic p-values (−log_10_P) for the SNPs in the MHC region that includes class I and class III non-classical MHC genes. Panel A has the −log_10_P for the univariate analysis, and panel B the −log_10_P adjusting for DRB1*15:01 in the model. Shades of red represent the r2 between the SNPs and the best marker, i.e. rs2516489.
(PDF)

Figure S3 DRB1*15:01-stratified analysis for the SNPs tagging the non-classical HLA LD haplotype. The y-axis lists the SNPs in positional order. The x-axis displays the respective Odds Ratios and 95% confidence intervals of these SNPs while analyzing with a univariate model: a) all individuals [cases: 5,091; controls: 9,595] (black color), b) DRB1*15:01 carriers [cases: 2,794; controls: 2,392] (green color), and c) non-carriers of DRB1*15:01 [cases: 2,367; controls: 7,204] (red color). Asterisks indicate associations with a p-value less than 1×10^−05^.
(PDF)

Figure S4 Analysis of amino acid residues in DRβ1. The univariate analysis results are in the first row. Each next one plots the −log10(p-value) of the amino acid positions conditioning on the amino acid residues if the previous rows. The solid black line marks the threshold of statistical significance in the study. The rows represent univariate analysis (A), conditioning on position 71 (B), conditioning on the above and position 74 (C), conditioning on the above and position 57 (D), conditioning on the above and position 86 (E).
(PDF)

Table S1 Statistically independent effects of the DRB1 locus considering four digit resolution alleles. In all cells Odds Ratios are listed. P-values are listed in parentheses for the forward stepwise regression. The order of the DRB1 alleles is according to the forward stepwise regression (primary analysis). The stopping rule was the residual DRB1 locus effect to have a p-value>1.0e^−05^. * For the regression-based methods the step number in which the allele was included in the model is displayed. Effect sizes (and p-values) per variants are for the respective step in the forward stepwise regression and for the final model in the least angle and forward stagewise regressions. ^$^ For the lasso and elastic net the Odds Ratios are displayed, since both methods provide the best solution. The largest value of *l_1_* regularization parameter such that error is within 1 standard error of the minimum *l_1_* regularization parameter was used to identify the best solution. Both the lasso and elastic net also identified *01:01 in their best solution, with OR of 0.99 and 0.97, respectively. This allele comes up in step 7 in all regression methods.
(DOC)

Table S2 Proof of statistical independence of rs2516489 and HLA-DRB1*15:01. The interaction term of the two variants cannot explain either of the two effects. Especially in the saturated model (both variants and the interaction term) the interaction term is not statistically significant, even at the nominal level. The stratification of the samples, based on HLA-DRB1*15:01 carrier status, reveals the effect of rs2516489 in both strata. The numbers listed are the Odds Ratio (OR), followed by the p-value. In all models principal components and dummy variable for studies were used as covariates.
(DOC)

Table S3 Cis-eQTL effects (p-value<0.05) of the SNPs in MICB-LST1 in PBMCs of MS subjects, CD4+ T cells of healthy individuals and CD14+ monocytes of healthy individuals. This table is included in the accompanied xls entitled “Supplementary_Table_3.xls”. None of the cis-eQTLs reached statistical significance (p-value<1×10^−5^).
(XLS)

Table S4 Association of the six identified DRB1 alleles and the amino acid changes in the four associated DRβ1 positions. Amino acids that predispose to MS susceptibility are indicated with bold. The rest are either protective or neutral. *DRB1* alleles in bold predispose to MS and the rest are protective.
(DOC)

Table S5 Imputation quality scores for the identified variants.
(DOC)

Text S1 Supplementary text explaining the chromatin states coloring scheme.
(DOC)
