# Supplementary figures and images for: Fine-Mapping the Genetic Association of the Major Histocompatibility Complex in Multiple Sclerosis: HLA and Non-HLA Effects
Source: PLoS Genet. 2013 Nov 21;9(11):e1003926. doi: 10.1371/journal.pgen.1003926 (PMC3836799; doi:10.1371/journal.pgen.1003926)

**SNPs**

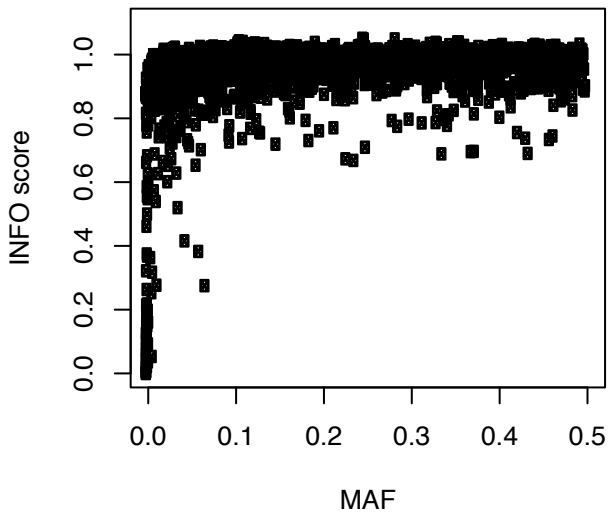

**2 digits HLA alleles**

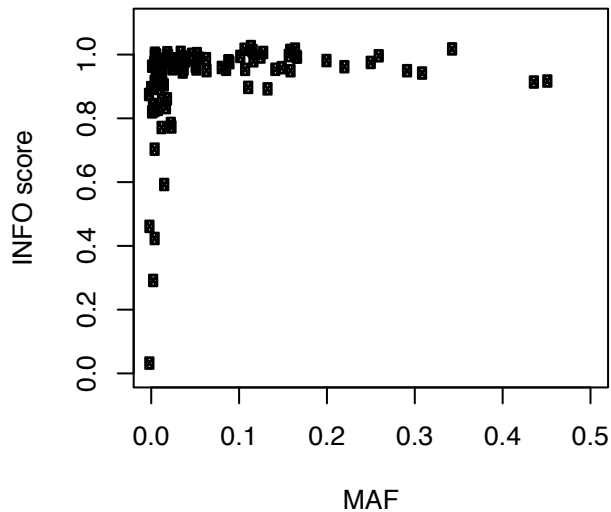

**4 digits HLA alleles**

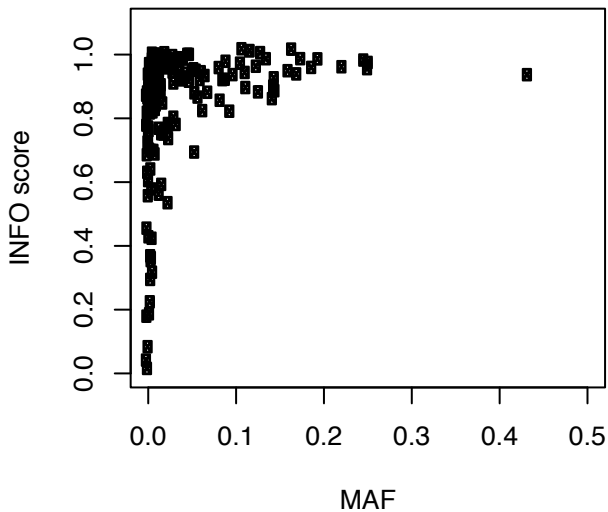

**Amino-acids**

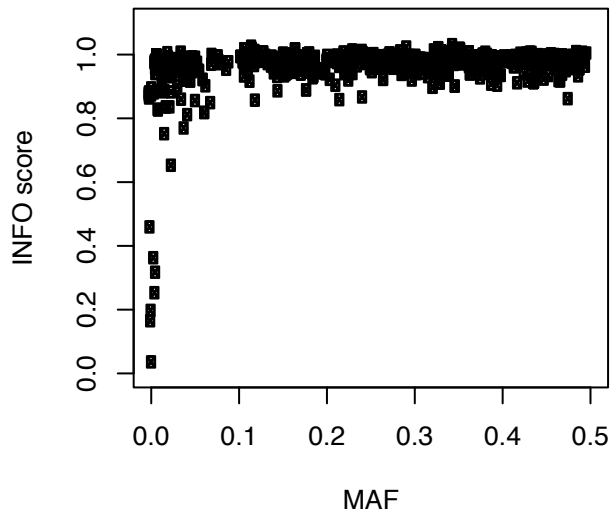

Supplement: Figure S1 — Scatter plots of minor allele frequency (MAF) and INFO score for the imputed variants. INFO score is an imputation quality metric and is defined as the ratio of the variance observed over the variance expected. On average genotyped SNPs have a value ∼1. (PDF) [file pgen.1003926.s001.pdf]

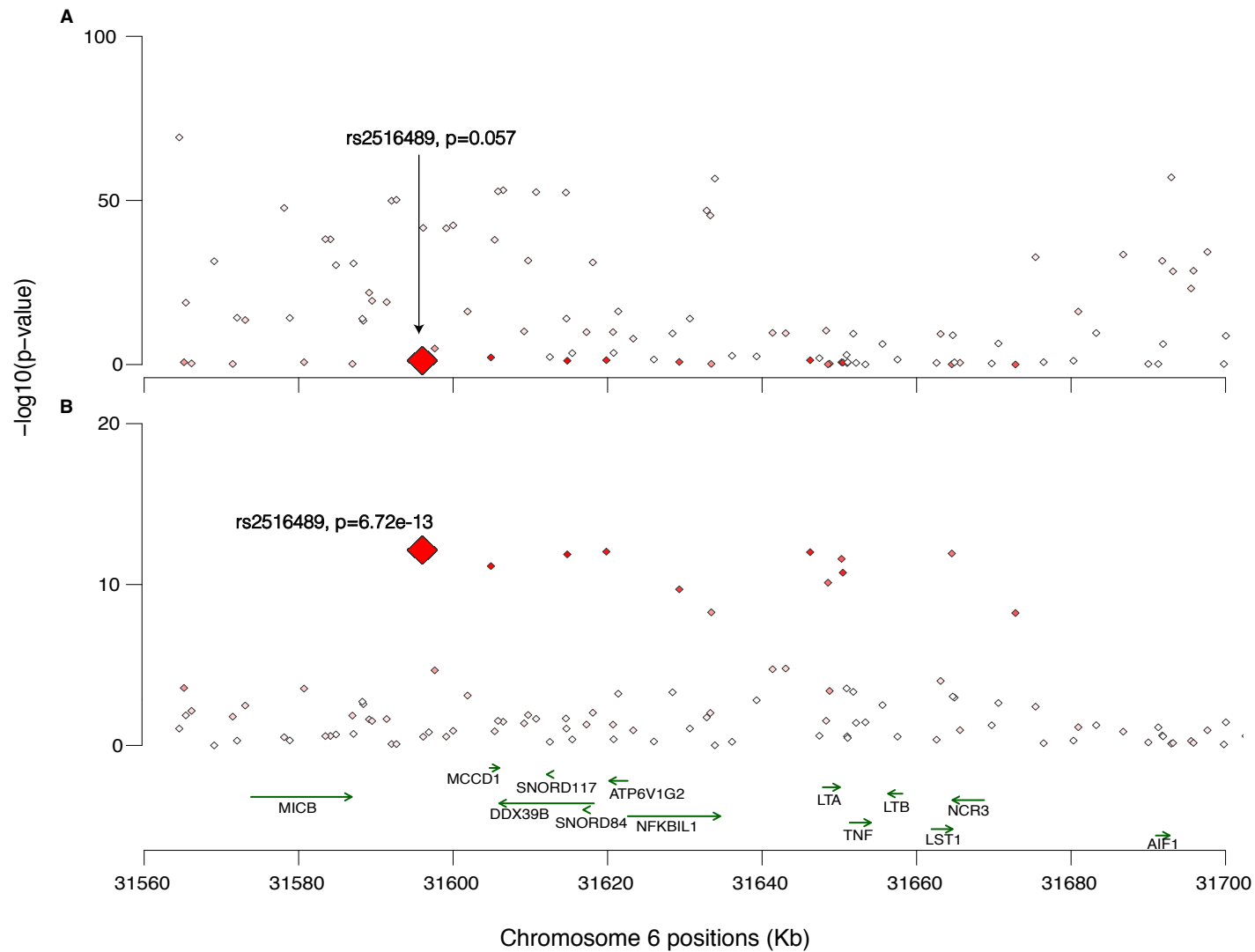

Supplement: Figure S2 — Regional associational plots for the non-classical HLA region spanning MICB-LST1. The figure displays the minus logarithmic p-values (−log10P) for the SNPs in the MHC region that includes class I and class III non-classical MHC genes. Panel A has the −log10P for the univariate analysis, and panel B the −log10P adjusting for DRB1*15:01 in the model. Shades of red represent the r2 between the SNPs and the best marker, i.e. rs2516489. (PDF) [file pgen.1003926.s002.pdf]

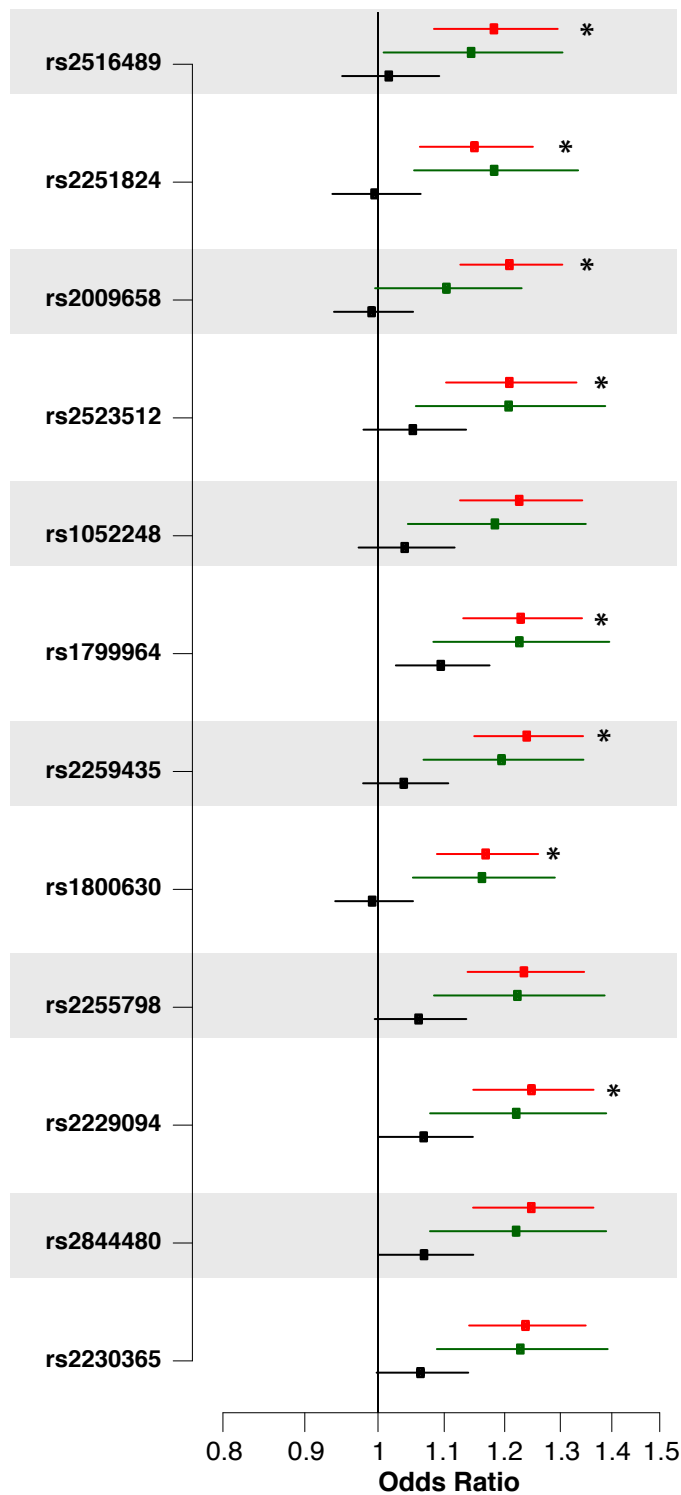

Supplement: Figure S3 — DRB1*15:01-stratified analysis for the SNPs tagging the non-classical HLA LD haplotype. The y-axis lists the SNPs in positional order. The x-axis displays the respective Odds Ratios and 95% confidence intervals of these SNPs while analyzing with a univariate model: a) all individuals [cases: 5,091; controls: 9,595] (black color), b) DRB1*15:01 carriers [cases: 2,794; controls: 2,392] (green color), and c) non-carriers of DRB1*15:01 [cases: 2,367; controls: 7,204] (red color). Asterisks indicate associations with a p-value less than 1×10^−05^. (PDF) [file pgen.1003926.s003.pdf]

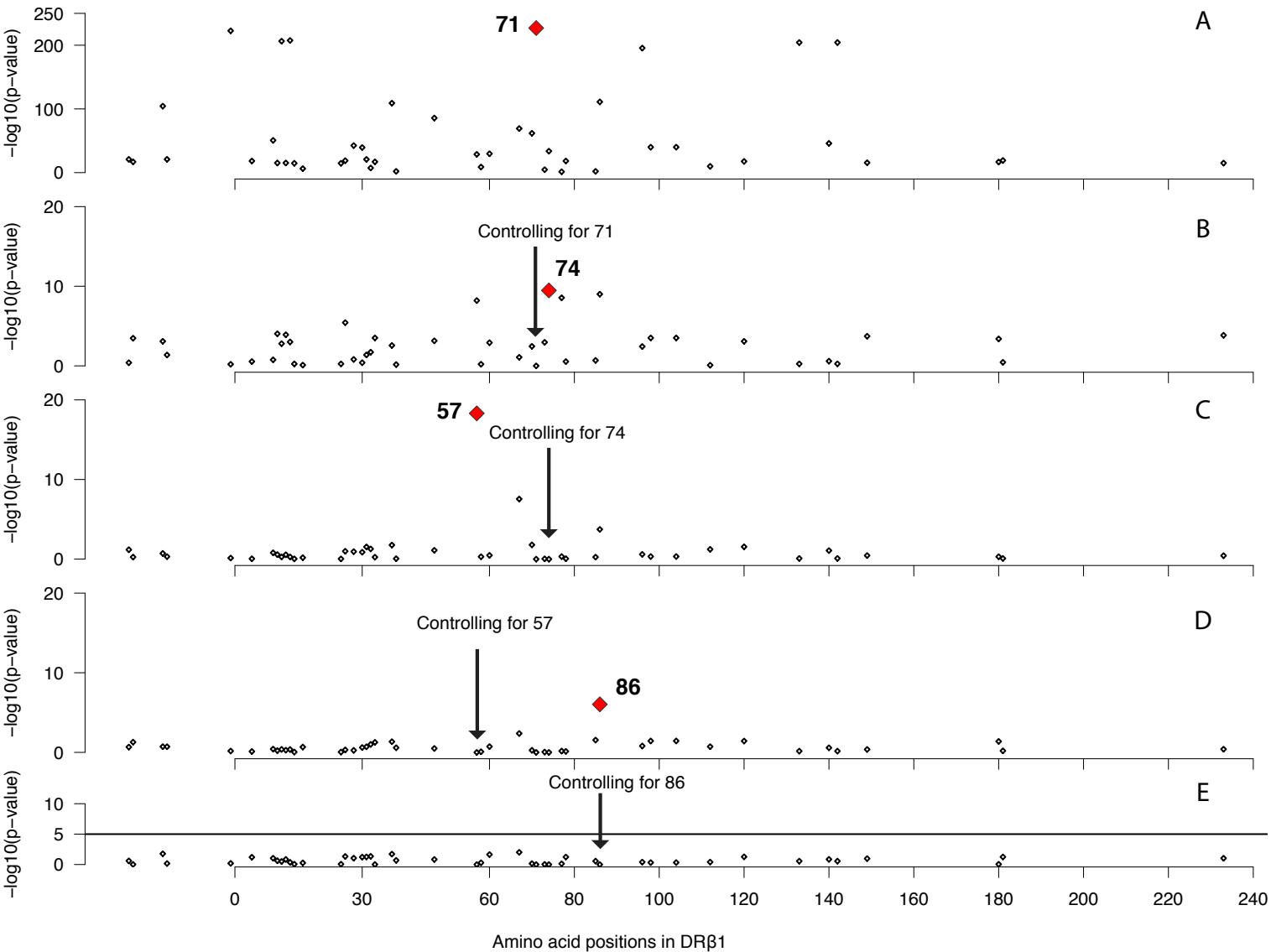

Supplement: Figure S4 — Analysis of amino acid residues in DRβ1. The univariate analysis results are in the first row. Each next one plots the −log10(p-value) of the amino acid positions conditioning on the amino acid residues if the previous rows. The solid black line marks the threshold of statistical significance in the study. The rows represent univariate analysis (A), conditioning on position 71 (B), conditioning on the above and position 74 (C), conditioning on the above and position 57 (D), conditioning on the above and position 86 (E). (PDF) [file pgen.1003926.s004.pdf]
