# Supplementary material for: Fine-Mapping the Genetic Association of the Major Histocompatibility Complex in Multiple Sclerosis: HLA and Non-HLA Effects
Source: PLoS Genet. 2013 Nov 21;9(11):e1003926. doi: 10.1371/journal.pgen.1003926 (PMC3836799; doi:10.1371/journal.pgen.1003926)
Supplement: Table S1 — Statistically independent effects of the DRB1 locus considering four digit resolution alleles. In all cells Odds Ratios are listed. P-values are listed in parentheses for the forward stepwise regression. The order of the DRB1 alleles is according to the forward stepwise regression (primary analysis). The stopping rule was the residual DRB1 locus effect to have a p-value>1.0e−05. * For the regression-based methods the step number in which the allele was included in the model is displayed. Effect sizes (and p-values) per variants are for the respective step in the forward stepwise regression and for the final model in the least angle and forward stagewise regressions. $ For the lasso and elastic net the Odds Ratios are displayed, since both methods provide the best solution. The largest value of l1 regularization parameter such that error is within 1 standard error of the minimum l1 regularization parameter was used to identify the best solution. Both the lasso and elastic net also identified *01:01 in their best solution, with OR of 0.99 and 0.97, respectively. This allele comes up in step 7 in all regression methods. (DOC) [file pgen.1003926.s005.doc]

| *DRB1* allele | Variable selection method | | | | |
| --- | --- | --- | --- | --- | --- |
|  | Forward Stepwise  Regression* | Lasso^$^ | Elastic Net^$^ | Least Angle Regression* | Forward Stagewise Regression* |
| **15:01* | Step 1: 2.92 (1.38e-234) | 2.68 | 2.50 | Step 1: 1.25 | Step 1: 1.25 |
| **03:01* | Step 2: 1.37 (9.37e-16) | 1.17 | 1.14 | Step 3: 1.04 | Step 3: 1.03 |
| **13:03* | Step 3: 2.66 (2.46e-15) | 1.68 | 1.59 | Step 2: 1.12 | Step 2: 1.12 |
| **04:04* | Step 4: 1.48 (3.22e-06) | 1.03 | 1.01 | Step 6: 1.01 | Step 6: 1.01 |
| **04:01* | Step 5: 0.81 (1.63e-04) | 0.91 | 0.89 | Step 4: 0.98 | Step 4: 0.98 |
| **14:01* | Step 6: 0.65 (7.06e-04) | 0.96 | 0.93 | Step 5: 0.99 | Step 5: 0.99 |
