# Supplementary material for: Fine-Mapping the Genetic Association of the Major Histocompatibility Complex in Multiple Sclerosis: HLA and Non-HLA Effects
Source: PLoS Genet. 2013 Nov 21;9(11):e1003926. doi: 10.1371/journal.pgen.1003926 (PMC3836799; doi:10.1371/journal.pgen.1003926)
Supplement: Table S2 — Proof of statistical independence of rs2516489 and HLA-DRB1*15:01. The interaction term of the two variants cannot explain either of the two effects. Especially in the saturated model (both variants and the interaction term) the interaction term is not statistically significant, even at the nominal level. The stratification of the samples, based on HLA-DRB1*15:01 carrier status, reveals the effect of rs2516489 in both strata. The numbers listed are the Odds Ratio (OR), followed by the p-value. In all models principal components and dummy variable for studies were used as covariates. (DOC) [file pgen.1003926.s006.doc]

|  | **rs2516489^T^** | **rs2516489^T^ * DRB1*15:01**  **interaction term** | **DRB1*15:01** |
| --- | --- | --- | --- |
| Univariate analysis | 1.07; 0.057 | - |  |
| rs2516489 and *DRB1*15:01* | 1.30; 7.68 x 10^-11^ | - | 3.02; 5.29 x 10^-243^ |
| rs2516489 * *DRB1*15:01* interaction term only | - | 2.43; 1.46 x 10^-60^ | - |
| rs2516489, *DRB1*15:01* and rs2516489 * *DRB1*15:01* interaction term | 1.23; 1.50 x ^-06^ | 1.09; 0.25 | 2.96; 3.54 x 10^-178^ |
| Univariate analysis for *DRB1*15:01* carriers | 1.23; 9.27 x 10^-04^ | - | - |
| Univariate analysis for *DRB1*15:01* non-carriers | 1.24; 5.10x 10^-07^ | - | - |
