# Supplementary material for: Fine-Mapping the Genetic Association of the Major Histocompatibility Complex in Multiple Sclerosis: HLA and Non-HLA Effects
Source: PLoS Genet. 2013 Nov 21;9(11):e1003926. doi: 10.1371/journal.pgen.1003926 (PMC3836799; doi:10.1371/journal.pgen.1003926)
Supplement: Table S4 — Association of the six identified DRB1 alleles and the amino acid changes in the four associated DRβ1 positions. Amino acids that predispose to MS susceptibility are indicated with bold. The rest are either protective or neutral. DRB1 alleles in bold predispose to MS and the rest are protective. (DOC) [file pgen.1003926.s008.doc]

|  | **71** | **74** | **57** | **86** |
| --- | --- | --- | --- | --- |
| ****15:01*** | **Ala** | **Ala** | **Asp** | **Val** |
| ****03:01*** | Lys | Arg | **Asp** | **Val** |
| ****13:03*** | Lys | **Ala** | **Ser** | Gly |
| ****04:04*** | Arg | **Ala** | **Asp** | **Val** |
| **04:01* | Lys | **Ala** | **Asp** | Gly |
| **14:01* | Arg | Glu | Ala | **Val** |
