# Supplementary material for: Fine-Mapping the Genetic Association of the Major Histocompatibility Complex in Multiple Sclerosis: HLA and Non-HLA Effects
Source: PLoS Genet. 2013 Nov 21;9(11):e1003926. doi: 10.1371/journal.pgen.1003926 (PMC3836799; doi:10.1371/journal.pgen.1003926)
Supplement: Table S5 — Imputation quality scores for the identified variants. (DOC) [file pgen.1003926.s009.doc]

| Variant | INFO score |
| --- | --- |
| *HLA-DRB1*15:01* | 0.9865 |
| *HLA-DRB1*03:01* | 1.0050 |
| *HLA-DRB1*13:03* | 0.8724 |
| *HLA-DRB1*04:04* | 0.7849 |
| *HLA-DRB1*04:01* | 0.8741 |
| *HLA-DRB1*14:01* | 0.7684 |
| *HLA-A*02:01* | 0.9840 |
| *HLA-DPB1*03:01* | 0.9029 |
| *HLA-B*37:01* | 0.9849 |
| *HLA-B*38:01* | 0.8419 |
| rs2844821 | 1.0105 |
| rs9277489 | 0.9959 |
| rs2516489 | 0.9851 |
| CHR6_31431006 | 0.9850 |
| Val86 DRβ1 | 0.8694 |
| Lys71 DRβ1 | 0.9383 |
| Arg71 DRβ1 | 0.9247 |
| Glu71 DRβ1 | 0.8712 |
| Ala71 DRβ1 | 0.9823 |
| Ala74 DRβ1 | 0.9331 |
| Gln74 DRβ1 | 1.0075 |
| Arg74 DRβ1 | 1.0068 |
| Leu74 DRβ1 | 0.9723 |
| Glu74 DRβ1 | 0.7970 |
| Asp57 DRβ1 | 0.9208 |
| Val57 DRβ1 | 0.9752 |
| Ser57 DRβ1 | 0.8079 |
| Ala57 DRβ1 | 0.8637 |
| Il95 HLA-A | 0.9892 |
| Leu95 HLA-A | 0.9774 |
| Val94 HLA-A | 0.9887 |
| Leu65 HLA-DPβ1 | 0.9681 |
| Ser99 HLA-B | 0.9856 |
