# Supplementary material for: Fine-Mapping the Genetic Association of the Major Histocompatibility Complex in Multiple Sclerosis: HLA and Non-HLA Effects
Source: PLoS Genet. 2013 Nov 21;9(11):e1003926. doi: 10.1371/journal.pgen.1003926 (PMC3836799; doi:10.1371/journal.pgen.1003926)
Supplement: Text S1 — Supplementary text explaining the chromatin states coloring scheme. (DOC) [file pgen.1003926.s010.doc]

**Supplementary material**

**Detailed chromatin states of the ENCODE data (GM12878**)

|  | Active promoter |
| --- | --- |
|  | Weak promoter |
|  | Inactive/poised promoter |
|  | Strong enhancer |
|  | Strong enhancer |
|  | Weak enhancer |
|  | Weak enhancer |
|  | Insulator |
|  | Transcriptional transition |
|  | Transcriptional elongation |
|  | Weak transcribed |
|  | Polycomb-repressed |
|  | Heterochromatin, low signal |
|  | Repetitive/CNV |
|  | Repetitive/CNV |

Detailed chromatin states for the NIH Epigenomics Roadmap (CD4 and CD8)

|  | TSS_poised |
| --- | --- |
|  | TSS_flanking_more_upstream |
|  | TSS_active |
|  | TSS_weak |
|  | TSS_flanking_downstream |
|  | TSS_flanking_more_downstream |
|  | Transcription |
|  | Transcription_weak |
|  | Enhancer-like_Genic |
|  | Enhancer-like_Genic_(short_genes) |
|  | Enhancer_weak_1 |
|  | Enhancer_weak_2 |
|  | Enhancer_active |
|  | Enhancer_active_with_weakK4me1_strong_K27ac |
|  | Enhancer_poised |
|  | Repressed_polycomb_weak |
|  | Repressed_polycomb |
|  | H3K9me3_K27me3 |
|  | Zinc_finger_genes_H3K36me3_K9me3 |
|  | Heterochromatin_at_repeats |
|  | Heterochromatin |
|  | Quiescent_1 |
|  | Quiescent_2 |
|  | Quiescent_3 |
|  | H3K9ac_low |
